# Decreased Concussion Incidence Following the Implementation of the Targeting Rules: An Updated Epidemiology of National Football League Concussions From 2017 to 2022

**DOI:** 10.7759/cureus.50997

**Published:** 2023-12-23

**Authors:** Jared M May, Hunter S Angileri, Daniel E McLoughlin, Madeline M Owen, Michael Terry, Vehniah Tjong

**Affiliations:** 1 Department of Orthopaedic Surgery, Northwestern University Feinberg School of Medicine, Chicago, USA

**Keywords:** nfl, return to sport, sports injury, concussion, football

## Abstract

The incidence of concussions in football, and the ensuing media attention, has garnered scientific investigation, prompted technological advances in protective gear, and altered the rules of the game, including the National Football League’s (NFL) “Targeting” rule, which began in 2018, but the impact of these changes is unclear. This study aims to describe the epidemiology of concussions that occurred in five NFL seasons from the 2017-2018 season through the 2021-2022 season and characterize positional differences in rate and games missed. There was a significant decrease (p = 0.02) in total concussions between the 2017-2018 season (102 concussions) and the remaining four seasons (average of 73.80 concussions per year), accounting for a 38% decrease. Offensive and defensive units had decreased concussion rates and average games missed per concussion. Defensive backs (10.46 per 1,000 athlete exposures (AEs)) and tight ends (10.69 per 1,000 AEs) had the highest concussion rates, and the defensive line had the highest average games missed per concussion at 3.97. The introduction of the “Targeting” rule and other rule changes in the NFL in 2018 correlated with a decrease in total concussions per year, total games missed due to concussion, and average games missed per concussion. Offense and defense experienced similar reductions in concussion incidence and severity. Overall, the updated epidemiology of NFL concussions suggests that the incidence of concussions has decreased; however, players continue to experience concussions that require them to miss multiple games.

## Introduction

The rising incidence of concussions in sports, and the media attention that followed, has garnered scientific investigation, prompted technological advances in protective gear, altered the rules of games, and spurred legislation. Due to the number of participants and the frequency and force of collisions, concussions in football are a public health concern with unknown long-term consequences, affecting participants at all levels [1,2]. Similarly, clinicopathological evaluation of former football players has revealed evidence of chronic traumatic encephalopathy in nearly all brains studied from former National Football League (NFL) and collegiate players and 21% of high school players, highlighting the importance of protecting athletes during play from head trauma [3].

The last two decades have seen numerous studies characterizing the epidemiology of concussions in the NFL. These studies have characterized factors influencing changes in concussion rates [4-10], including positional differences [4,5,11,12]. Specifically, in 2004, the first study examining the epidemiology of concussions among NFL players was published by the Mild Traumatic Brain Injury Committee, reporting a rate of 0.41 concussions per game in the seasons between 1996 and 2001, with the greatest relative risk for quarterbacks, wide receivers, and tight ends [4]. Additional works by the committee characterized the positions most susceptible to repeat concussions [11] and concussions involving seven or more days missed, of which quarterbacks, the return unit on special teams, and defensive secondary were most susceptible [12].

In their follow-up study regarding the next six seasons (2002-2007), the rate of concussions per game overall decreased to 0.38, and rates of concussions in quarterbacks and wide receivers decreased, while the rate for tight ends increased [5]. Further studies over the next 10 seasons have suggested that the concussion rate per game ranges from 0.59 to 0.66 [6-10]. Factors associated with more games missed include playing quarterback, being involved with more plays per game, and having a history of multiple concussions [13]. Other studies have focused on how the incidence of concussions varies over the course of the season, with one study reporting an increase in concussions in the latter half of the season [14] while another noted no significant difference in games deemed “important” or “unimportant” in weeks 13-16 of the season, based on a team’s eligibility for the playoff [15].

With the increased awareness of concussion risk and its subsequent long-term health consequences, the NFL faced pressure to improve player safety and further reduce concussion risk. Their changes not only included the adoption of new helmet technology but also significant updates to contact rules [16,17]. Beginning in the 2018 season, the “Targeting” rule was enacted, penalizing hits with or directed at the head. Additionally, changes to special teams play have aimed to reduce the force and frequency of collisions on kickoffs [18]. While these changes have decreased the rate of concussions in their initial seasons [19], it is unclear how the epidemiology of concussions has evolved in the following years, including changes to the number of games missed per concussion, positional differences in time missed, and the addition of a 17th regular season game in 2021.

The present study addresses this gap in the literature by describing the epidemiology of concussions across the last five NFL seasons. The aim of the current study was to determine changes in concussion incidence over the study period and to highlight positional differences in rates and games missed. We hypothesized that the introduction of recent rule changes would be associated with a reduction in concussion incidence and time loss due to concussion. We also hypothesized that the addition of a 17th regular season game would increase the total number of concussions, but not impact the rate. Additionally, we anticipate that positional differences in concussion rate and severity will persist.

This article was previously presented as a meeting abstract at the 2023 American Orthopaedic Society for Sports Medicine annual meeting on July 15, 2023 [20].

## Materials and methods

The current study was a descriptive epidemiological study that retrospectively reviewed concussions across five NFL seasons, from the 2017-2018 season through the 2021-2022 season. Concussion data extraction methods conformed to methods outlined in prior studies on the epidemiology of NFL reportable injuries [21], defined as necessitating physician referral or care and resulting in missed competition. Concussion data were extracted from a publicly available injury report (https://www.pro-football-reference.com/players/injuries.htm) [22] and cross-referenced with other publicly available sources (https://www.nfl.com/injuries/ and https://www.espn.com/nfl/injuries) [23,24] to verify injury data, which are updated weekly. The study did not require an Institutional Review Board (IRB) approval due to the database’s public presentation. This study only included concussions that resulted in one or more missed games. Due to the indistinguishable timing of injuries that occurred prior to the first regular season game, concussions that occurred in preseason games, training camp, or otherwise prior to week one of the regular season were excluded from the study.

Statistical analysis

In accordance with prior studies that analyzed injury rates in athletes, concussion incidence was normalized per 1,000 athlete exposures (AEs), with one AE equal to one game per athlete [10,21]. Total injuries represent the total number of concussions over the given time period for the indicated unit or position group. Number of games played represents the total number of games played over the given time period.

$AE = \frac{Total Injuries * 1,000}{(53 Player Roster Size)*(Number of Games Played)}$

Data analysis was conducted using Microsoft Office Excel (Microsoft Corporation, Redmond, WA). Single-factor ANOVA was used to compare categorical variables, with statistical significance set to p ≤ 0.05. Sub-analysis was performed by year, game unit, position type, and game week.

## Results

Total incidence

**Figure 1:**
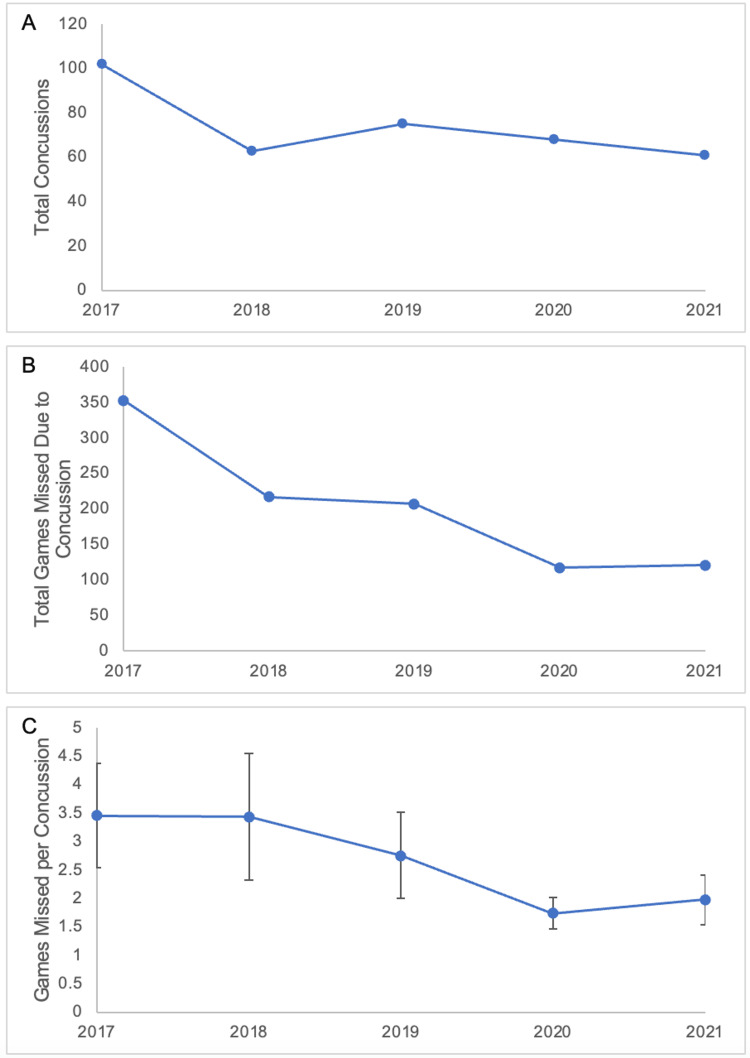
Total concussions and games missed from 2017 to 2022 A: Total concussions per season. B: Total games missed due to concussion. C: Games missed per concussion per season with 95% confidence intervals.

There were 1,275 regular season and postseason games in the five NFL seasons between 2017-2018 and 2021-2022. Over those five seasons, there were a total of 368 concussions that resulted in at least one game missed for a rate of 0.30 concussions per game, or 7.04 concussions per 1,000 AEs. Descriptively, the 2017-2018 season had the highest number of total concussions (102), with an average of 73.80 concussions for the five seasons studied. A one-way ANOVA revealed that there was a statistically significant decrease (38%, p = 0.02) in total concussions between the 2017-2018 season and the four seasons between 2018-2019 and 2021-2022 (average of 66.75) (Figure 1A).

**Figure 2:**
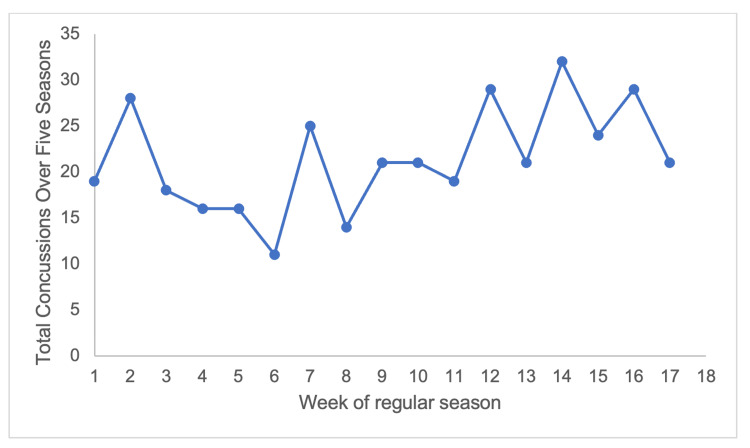
Concussion incidence varies by game week Concussion total by week of regular season.

During these five years, there were 1,014 games missed due to concussion, for an average of 2.76 games missed per concussion and 0.06 games missed due to concussion per 1,000 AEs. The 2017-2018 season also had the highest total amount of games missed due to concussion and the highest average games missed per concussion at 3.46. Following the 2017-2018 season, there was a 53% decrease in the total games missed due to concussion (Figure 1B, p = 0.05) and a 38% decrease in the average games missed per concussion (Figure 1C, p = 0.34). There was a significantly higher concentration of concussions during the last one-third of the regular season (p = 0.03) (Figure 2).

Incidence by unit

**Figure 3:**
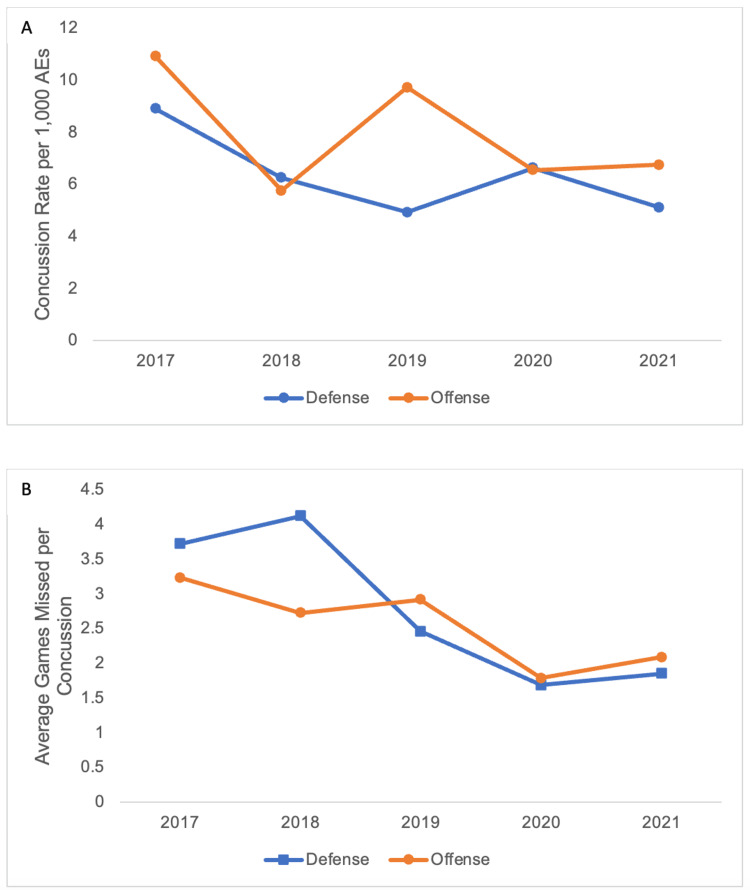
Concussion rate and average games missed decreased over the study period A: Unit-specific concussion rate per 1,000 athlete exposures (AEs). B: Average games missed per concussion.

The 2017-2018 season had the highest injury rate per 1,000 AEs and the greatest average games missed per concussion in both offensive and defensive units. Both units experienced a decreased concussion rate (Figure 3A) and average games missed per concussion (Figure 3B) in the subsequent four seasons. Comparing the 2017-2018 season with the subsequent four seasons, defensive players experienced a significantly decreased rate of concussions per 1,000 AEs (p = 0.04); however, offensive players did not.

Incidence by position

**Figure 4:**
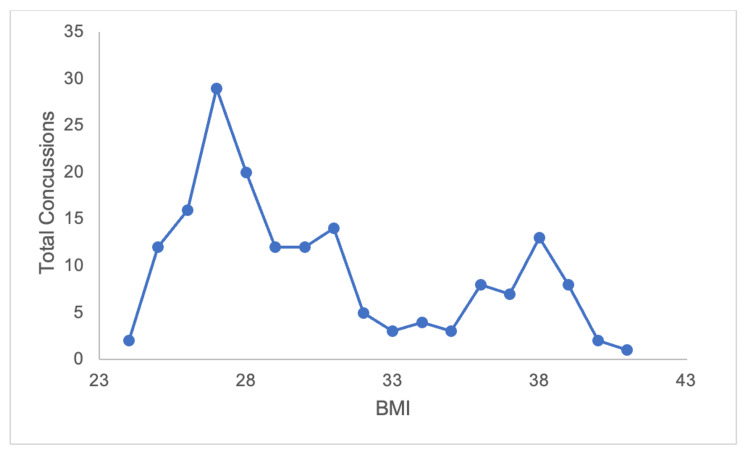
Concussion incidence varies by player's BMI Total five-year concussions by BMI.

Defensive backs (10.46 per 1,000 AEs) and tight ends (10.69 per 1,000 AEs) had the highest concussion rates by position groups over the five years studied. Defensive line players (3.70 per 1,000 AEs) had the lowest concussion rate. The defensive line had the highest average games missed per concussion (3.97) over these five seasons and defensive backs had the lowest average games missed per concussion (2.20). This difference was significant (p = 0.02) between these two positions; however, there was no significant difference between positions overall (p = 0.19). Offensive players had slightly higher concussion rates compared to the defense (7.83 per 1,000 AEs versus 6.28 per 1,000 AEs). However, defensive players had slightly more average games missed per concussion at 2.88 versus 2.65 games missed per concussion for the offense; this was not significant (p = 0.54) (Table 1). When stratifying the incidence of concussions by body mass index (BMI), there was a global maxima in players with a BMI of 27 and a local maxima in players with a BMI of 38 (Figure 4).

**Table 1:** Concussion rate by position in AE and average games missed Position-specific concussion rate per 1,000 athlete exposures (AEs) and games missed per concussion between the 2017-2018 and 2021-2022 National Football League seasons. The p-values compare the mean for each position group versus the overall mean games missed for all concussions. The p-values for offense versus defense compare the two units to each other.

Position	Total concussions	AEs	Concussion rate per 1000 AEs	Average games missed per concussion (95% CI)	p-value
Defensive line	36	9728	3.70	3.97 (1.76)	0.07
Linebacker	43	8512	5.05	3.37 (1.29)	0.30
Offensive line	69	8512	8.11	2.30 (0.73)	0.33
Quarterback	13	2432	5.35	2.77 (1.71)	0.99
Running back	21	4864	4.32	2.90 (1.50)	0.85
Defensive back	89	8512	10.46	2.20 (0.61)	0.18
Tight end	39	3648	10.69	3.36 (1.33)	0.33
Wide receiver	54	6080	8.88	2.46 (0.71)	0.57
Defense	168	26752	6.28	2.88 (0.60)	0.54
Offense	200	25536	7.83	2.65 (0.45)	0.54

## Discussion

The current study was an updated epidemiology of concussions across the previous five NFL seasons, from 2017 to 2022. Results suggest that there was a decrease in total concussions, total games missed due to concussions, and average games missed per concussion following the 2017-2018 season. The downward trends were also seen across both offensive and defensive units. Furthermore, defensive backs and tight ends had the highest concussion rates, while the defensive line had the highest average games missed per concussion. The updated analysis suggests that the introduction of rule changes after the 2017-2018 season was associated with a reduction in concussion incidence and length of games missed. However, concussions resulting in multiple games missed continue to occur, and player characteristics such as position cause disproportionate rates of injuries.

In addition to impacting the immediate health of the over 1,700 active NFL players, traumatic head injuries also have significant and detrimental long-term neural and cognitive outcomes. Repetitive head trauma has been associated with the progressive neurodegenerative disease of chronic traumatic encephalopathy (CTE), which is characterized by dementia, memory loss, aggression, confusion, and depression [25]. In 2017, Mez et al. reported that 99% of donated brains from former NFL players demonstrated neuropathological signs of CTE [3].

With the increasing awareness of the long-term consequences of concussions, the NFL began implementing changes to both the rules of the game and protective equipment to improve player safety. Perhaps no recent rule change has garnered as much attention as the “targeting” rule, which restricted forcible hits to the head or with the head, aiming to protect offensive players from hits by the defense, and defensive players from their own hits. Most recently, in the 2022 season following an on-field collision involving Miami Dolphins quarterback Tua Tagovailoa, ataxia was added as a symptom necessitating immediate removal from play to the sideline concussion diagnosis protocol [26]. Given the burden that concussions can have on a player’s safety and long-term quality of life, it is important to analyze how these recent changes have impacted the epidemiology of concussions.

Across the five NFL seasons from 2017 to 2022, and since the enaction of the “targeting” rule and other changes, there was a significant decrease of 38% in the incidence of concussions resulting in at least one game missed (Figure 1A). This finding is consistent with the findings of Mack et al., who also reported a decrease in concussion incidence in the season immediately following the implementation of the targeting rule [19]. This suggests that the rule change, in combination with continually evolving protective gear technology, may contribute to the continued reduction in the risk of concussions in NFL players. Additionally, this study is the first to report the total games missed due to concussions and the number of games missed per concussion in the NFL. Both parameters decreased after the 2017-2018 season, which suggests that concussions that are still occurring are of reduced severity. Taken together, the decreases highlight that the rule change may have altered player behavior, encouraging them to decrease the force of hits and avoid the head.

In the 2021-2022 season, the NFL added an additional game to each team’s schedule. This change was a major player objection in the 2020 players union collective bargaining agreement, and there was fear that it would proportionally increase the total number of injuries, including concussions [27]. However, despite the additional game, the 2021-2022 season had the lowest total concussions (61) resulting in at least one game missed, indicating that an increase in games did not have a gross impact on total concussions, though only one season of data exists. Future studies should explore this impact on other injuries in NFL players. Interestingly, we observed an increase in concussions in the last one-third of the regular season. The reason for the increased incidence is unclear; however, our results agree with Teramoto et al., who previously reported an increase in concussions in the second half of a season, citing physical/mental fatigue and decreased temperatures as possible causes [14].

It is also important to understand how athlete characteristics such as unit, position, and BMI may influence changes in concussion epidemiology. By position, the largest decreases in concussion rate and games missed were by defensive players, while offensive players demonstrated a fluctuating concussion rate but a slight decrease in average games missed. This suggests that recent rule changes and technological advances have a greater impact on defensive players than offensive players. It is hypothesized that the “targeting” rule may have protective effects for the defensive player delivering a hit, potentially discouraging them from leading with their own head when initiating contact. Defensive backs and tight ends had the highest rate of concussion of any position, emphasizing the high-impact nature of these positions and the frequency of collisions, which aligns with previous findings by Mack et al. [19]. The defensive line had the lowest concussion rate, but also the greatest number of games missed, which does stand in contrast to previous findings that demonstrate quarterbacks having the greatest average games missed [13]. This may be due to the frequency of impact defensive line players experience, where repetitive microtrauma may cumulate in a concussion, and the NFL’s increased emphasis on protecting quarterbacks in recent years.

There are several limitations that should be considered when interpreting the results of this study. With concussion remaining a clinical diagnosis that combines recognition of subtle neurological changes and patient-reported symptoms, and with player motivation for non-disclosure, the current study's results may not represent the true incidence of NFL concussions [28,29]. Additionally, the study only analyzed concussions that resulted in at least one game missed and did not capture concussions that allowed athletes to return to play without missing a game [4]. Furthermore, as is common among other retrospective studies of sports injuries, the data lacked specific details about injury diagnoses and medical clearance, which may not be consistent with the return to play given the combination of medical and non-medical factors involved with the return to competition. It is also unclear if the captured concussions occurred in game or practice scenarios. The “targeting” rule and other rule changes may not have had a significant impact on concussions that occurred during practice, as players are less likely to deliver penalizable hits during practice to their own teams. However, concussions do still occur during practice, as Mack et al. have reported previously on the 2015-2019 seasons and found that 20% of total concussions occurred outside of NFL games [19]. In addition, the severity of concussions could not be directly assessed using the current database, which is a limitation common among other retrospective studies. Instead, the number of games missed was used as a proxy for severity, though it is understood that severity does not necessarily correlate with time missed [13]. Despite these limitations, we believe that the data presented in this study are the best representative of the epidemiology of concussions that occurred during the NFL seasons from 2017 to 2022 without direct collaboration from the NFL.

## Conclusions

Following the introduction of the “Targeting” rule and altered kickoff rules after the 2017-2018 NFL season, NFL players experienced a decrease in total concussions per year, total games missed per concussion, and games missed per concussion. Furthermore, offensive and defensive units experienced similar reductions in concussion incidence and severity, but positional differences in concussion rate and severity still exist and offensive unit concussion incidence and severity did not decrease as much as the defensive units. Defensive backs and tight ends are at the greatest risk of experiencing a concussion that results in at least one game missed. Overall, the study suggests that efforts to protect professional football players from concussions have been successful; however, players continue to experience concussions that require them to miss multiple games. Insights from the updated epidemiology of concussions should be used by the NFL to continue to improve player safety and to lead targeted initiatives to minimize harm to players in higher-risk groups.
